# Exploring the Immune Landscape of ccRCC: Prognostic Signatures and Therapeutic Implications

**DOI:** 10.1111/jcmm.70212

**Published:** 2024-11-18

**Authors:** Minjie Pan, Xinchi Xu, Dong Zhang, Wei Cao

**Affiliations:** ^1^ Department of Urology The Affiliated Changzhou Second People's Hospital of Nanjing Medical University Changzhou Jiangsu China; ^2^ The State Key Lab of Reproductive; Department of Urology The First Affiliated Hospital of Nanjing Medical University Nanjing China

**Keywords:** clear cell renal cell carcinoma, immunotherapy, machine learning, prognosis, signature

## Abstract

The tumour immunological microenvironment is involved in the development of clear cell renal cell carcinoma (ccRCC). Nevertheless, the role of the immunological microenvironment in ccRCC has not been thoroughly investigated. In this study, we combined six ccRCC cohorts into a large cohort and quantified the expression matrix into 53 immunological terms using the ssGSEA algorithm. Five immune terms related to prognosis were screened through 1000 iterations of L1‐penalised (lasso) estimation and Cox regression analysis for immune‐related risk score (IRS) calculation. The IRS showed satisfactory prognosis prediction efficacy in ccRCC. We then compared the clinical and genomic characteristics of two IRS subgroups. Patients with low IRS showed a high level of tumour mutational burden (TMB) and a low level of copy number variation (CNV), indicating that low IRS group patients have a higher probability of responding to immunotherapy. We employed TIDE and subclass mapping analyses to corroborate our results, and the findings demonstrated that patients with a low IRS had a significantly greater percentage of immunotherapy response. According to the Genomics of Drug Sensitivity in Cancer (GDSC), patients with a high IRS had a decreased IC50 for sunitinib, which is the first‐line treatment for ccRCC patients. As a result, the immune characteristics of the microenvironment of ccRCC tumours have been explored, and a signature has been constructed. Analysis demonstrated that our signature could effectively predict prognosis and immunotherapy response rate.

## 1 Introduction

Kidney cancer, the most lethal malignancy of the urogenital system, is also the ninth most common cancer globally [1]. Clear cell renal cell carcinoma (ccRCC) is the most prevalent and deadliest subtype, characterised by high aggressiveness, metastatic potential and a significant mortality rate [2]. Immunotherapy has emerged as a promising cancer treatment by enhancing the patient's immune system, with immune checkpoint inhibitor (ICI) therapy notably advancing ccRCC treatment [3]. Nevertheless, the response to immunotherapy varies widely between individuals with ccRCC. To overcome the limitations of current treatments, new therapeutic targets must be explored. High‐throughput sequencing has facilitated a more precise understanding of epigenetic changes in ccRCC, offering new directions for research and treatment. These epigenetic markers can potentially be used for early diagnosis, prognostic evaluation and targeted therapy in ccRCC.

Multiple factors contribute to tumour initiation and progression. The process of tumour angiogenesis is necessary for tumour proliferation and metastasis and plays a crucial role in ccRCC4 prognosis [4]. The interplay between stromal cells, immune cells, tumour cells and other non‐cellular components in the tumour microenvironment (TME) has a role in tumour development and influences immunotherapy responses [5]. Immune cells are composed of many subpopulations of cells that differentiate from haematopoietic stem cells and perform crucial roles in immunological responses and TME [6]. Through intercellular communication and cytokine signal transduction, diverse immune cells regulate and eliminate tumour cells to preserve tissue homeostasis. As one of the main components of TME, density of mast cells is elevated in almost all tumour types and studies have revealed that it can be used as target for immunotherapy of various tumours including ccRCC [7]. In addition, polymorphisms in the IL‐13 and IL‐4R have also been linked to RCC development [8]. The frequency of variant alleles that enhance IL‐4 signalling was significantly associated with an elevated risk of ccRCC [9]. Additionally, several different types of patients with tumour have benefitted from PD‐1 and PD‐L1 ICI therapy and various tumours have shown remarkable objective responses to the treatment [10]. Immunotherapy is a treatment option for ccRCC because it manipulates the patient's immune system to attack the cancerous cells.

In this study, the prognostic significance of immunological terms in ccRCC was evaluated using a bioinformatics approach. Six independent ccRCC cohorts' RNA‐seq data and clinical information were collected via single‐sample gene set enrichment analysis (ssGSEA) to quantify immune infiltration and immune response. According to L1‐penalised estimation and Cox regression algorithm, five significant immune terms were screened to create a clinical prognostic signature and calculate immune‐related risk score (IRS). Then, a series of statistical analyses, including gene set enrichment analysis (GSEA), Kaplan–Meier survival analysis, immune signature analysis, tumour mutation burden (TMB) analysis, mutation analysis and immunotherapy analysis, were carried out to detect the clinical signature and immune response of different IRS groups. Based on these findings, we can learn more about immune infiltration and immune response in ccRCC and develop individualised treatment plans.

## 2 Materials and Methods

### 2.1 Data Retrieval and Processing

After a comprehensive investigation, six cohorts (TCGA, ICGC, GSE73731, GSE40435, E‐MTAB‐1980, CTPAC) with available expression matrix data and complete clinical information were recruited. TCGA‐KIRC data were obtained from (TCGA) database, ICGC‐RECA‐EU data were obtained from ICGC database, E‐MTAB‐1980 data were obtained from ArrayExpress database, and CTPAC RNA‐seq data were obtained from Clinical Proteomic Tumour Analysis Consortium (CTPAC) database. Two expression profiles (GSE73731 and GSE40435), along with their corresponding clinical features, were retrieved from the Gene Expression Omnibus (GEO) based on the following selection criteria: (1) the platform file contains more than 45,000 probes to ensure comprehensive gene symbol coverage; (2) all probe values for each sample are greater than zero; (3) complete clinical information is available, including survival time and status; and (4) each dataset includes a sample size of more than 100 patients.

Prior to applying the ‘ComBat’ algorithm, RNA‐seq data were normalised as Transcripts Per Million (TPM) to ensure comparability with microarray data. This normalisation step was essential for accounting for differences in sequencing depth and gene length across samples. Missing values in the datasets were addressed through statistical imputation techniques to avoid bias in subsequent analyses [11, 12]. Following these preprocessing steps, the ‘ComBat’ algorithm, as described by Johnson et al. [13], was employed to correct for potential batch effects across different cohorts, reducing non‐biological variability while preserving the biological signals. This method was chosen for its robustness in adjusting for batch effects even in small sample sizes, making it ideal for our study's datasets. Finally, the expression profile of 47 melanoma patients who had a positive response to immunotherapies was collected from the known literature and was used for the prediction of patients' immune responses to immunotherapy [14].

### 2.2 Functional Pathway Analysis and ssGSEA

Gene Set Enrichment Analysis (GSEA) was performed using the R clusterProfiler package, while single‐sample GSEA (ssGSEA) was conducted with the R GSVA package (version 4.0.2). The Hallmark gene set (MSigDB) served as the reference for examining differences in oncogenic pathways. The gene sets used to quantify 53 immunological terms were published on Figshare (https://figshare.com/articles/dataset/Immune_set/14286632).

### 2.3 Development of Immune‐Related Risk Score (IRS) Signature

TCGA‐KIRC cohorts were chosen as the training cohort, while additional datasets were utilised as validation cohort due to their equal number of samples. Prognosis‐related immune terms were initially identified using univariate Cox analysis with a *p*‐value threshold of < 0.01. To reduce dimensionality, L1‐penalised (lasso) estimation was applied across over 1000 iterations [15]. Finally, immune terms that appeared more than 50 times were included in the multivariate Cox regression model for IRS computation using the following formula: Immune riskscore (IRS) = coef1*term1 + coef2*term2 + coef3*term3 +…+ coefN*termN. Logistic regression for the best cutoff value was conducted using R SimDesign and tdROC packages. The prognostic significance of the signature was assessed utilising the Kaplan–Meier and ROC survival analysis curves.

### 2.4 Cell Culture and Stable Cell Line Construction

The human ccRCC cell lines, 786‐O and Caki‐1, along with the human kidney proximal tubule epithelial cell line HK2, were maintained in RPMI‐1640 medium supplemented with 10% fetal bovine serum (FBS) and 1% penicillin–streptomycin at 37°C in a humidified atmosphere containing 5% CO₂. Lentiviral shRNA vectors targeting CPA4 and a control vector were purchased from TsingkeBiotechnologyCo.,Ltd. The plasmids were then co‐transfected with second‐generation packaging plasmids (psPAX2 and pMD2.G) into 293 T cells using PEI. At 48 h post‐transfection, the culture medium containing viral particles was harvested, filtered through a 0.45 μm pore size filter to remove cellular debris, and concentrated using ultracentrifugation at 100,000 g for 2 h. The viral concentrate was then used to infect 786‐O, Caki‐1 cells. The medium was replaced with fresh RPMI‐1640 containing 2 μg/mL puromycin to select for stably transduced cells over a period of 14 days, with medium changes every 2 days. The target sequence of sh‐CPA4 were: GCGTATGACAACGGCATCAAA.

### 2.5 Quantitative Real‐Time PCR

Total RNA was extracted using TRIzol reagent (Invitrogen) according to the manufacturer's instructions and then was reverse transcribed into cDNA. Quantitative real‐time PCR (qPCR) was performed using SYBR Green PCR Master Mix (Applied Biosystems). The expression levels of CPA4 were normalised to GAPDH. Primer sequences used were as follows: CPA4 forward, 5'‐AGGTGGATACTGTTCATTGGGG‐3'; CPA4 reverse, 5'‐TTGCTGATCTCGTCTCCATTTC‐3'; GAPDH forward, 5'‐GGAGCGAGATCCCTCCAAAAT‐3'; GAPDH reverse, 5'‐ GGCTGTTGTCATACTTCTCATGG‐3'.

### 2.6 CCK‐8 Assay

Cell viability was assessed using the Cell Counting Kit‐8 (Meilunbio). Cells were seeded in 96‐well plates at a density of 1000 cells per well and incubated for 0, 24, 48 and 72 h. At each time point, CCK‐8 solution was added, and the plates were incubated for an additional 2 h. Absorbance was measured at 450 nm using a microplate reader.

#### 2.6.1 Colony Formation Assay

For colony formation assays, 500 cells were seeded in six‐well plates and cultured for 14 days. Colonies were fixed with methanol, stained with 0.1% crystal violet, and counted under a microscope.

### 2.7 Transwell Assay

Migration and invasion assays were conducted using Transwell permeable supports (Corning) with 8.0 μm pore size. For migration assays, 1 × 10^4^ cells were seeded into the upper chamber lined with non‐coated membranes. For invasion assays, the upper chamber was coated with Matrigel (BD Biosciences) to simulate the extracellular matrix barrier. In both assays, cells were seeded in serum‐free medium, while medium containing 20% FBS was placed in the lower chamber to serve as a chemoattractant. After incubation for 24 h, cells that had migrated or invaded through the pores were fixed with methanol, stained with 0.1% crystal violet and counted under a microscope.

### 2.8 Tumour Genomics Features Between Two IRS Groups

TMB represents the mutations number per megabase (mt/Mb). Somatic mutation data used to calculate TMB were obtained from cBioPortal database. Maftools was utilised to investigate significantly altered genes (*p*‐value < 0.05) that differed among two groups as well as gene mutations interaction impact. Copy number variation (CNV) analysis was applied using GISTIC_2.0 tool. Copy number gain and loss burdens were computed at focal and arm levels utilising GISTIC 2.0 output files. The mRNAsi and the mDNAsi were obtained from transcriptomic data, EREG‐mRNAsi and EREG‐mDNAsi were estimated utilising one‐class logistic regression machine learning (OCLR) machine‐learning algorithm [16]. Furthermore, immunological cells levels and estimated cells in tumour tissue were quantified with R software's estimate package.

### 2.9 Targeted Therapy and Immunotherapy Response Prediction

Tumour Immune Dysfunction and Exclusion (TIDE) is an algorithm that accurately models immune evasion and predicts immunotherapy response. Patients with a TIDE score > 0 were considered non‐responders, while those with a TIDE score < 0 were considered responders. The subclass mapping technique was utilised to analyse the commonalities in immunotherapy response among 47 immunotherapy‐responsive patients and two IRS‐ccRCC patient groups. Moreover, using the Genomics of Drug Sensitivity in Cancer (GDSC) dataset, we determined the half maximal inhibitory concentration (IC50) of 12 commonly used targeted therapy drugs to forecast treatment sensitivity using the R pRRophetic package.

### 2.10 Statistical Analysis

The R statistical language was utilised for all studies. All tests were two‐tailed, and a *p*‐value < 0.05 was considered significant. The independent sample *t*‐test was employed to analyse continuous variables with a normal distribution, with values reported as the mean ± standard deviation. For comparing continuous variables with skewed distributions, the Wilcoxon rank‐sum test was employed.

## 3 Results

### 3.1 Combination of Data and Quantification of Immune Terms

**FIGURE 1:**
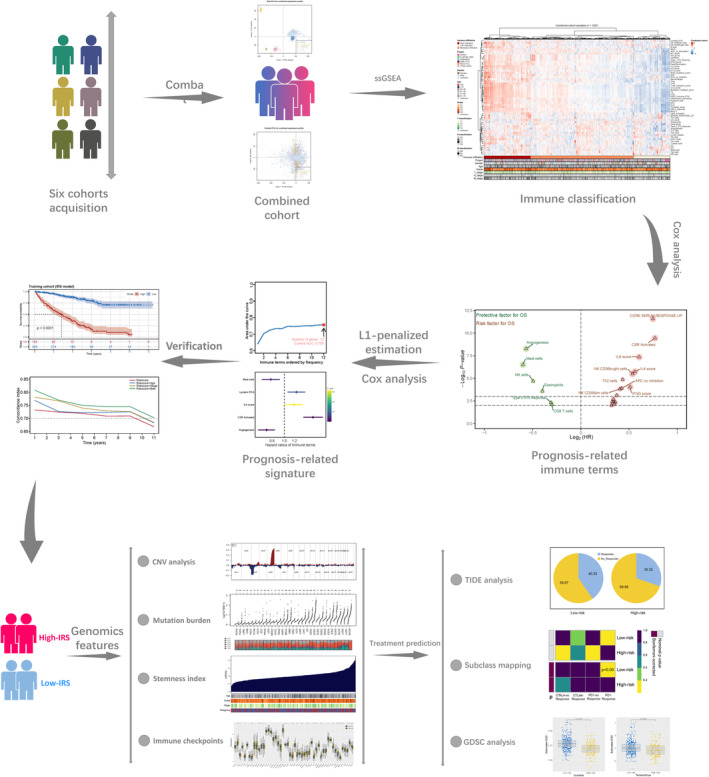
The flowchart of the analysis. Six independent ccRCC cohorts were randomly selected and combined into a single large cohort using the R sva package for subsequent analysis. The R ssGSEA package was then applied to quantify 53 immune‐related terms from the gene expression matrix. Through L1‐penalised estimation and Cox regression analysis, five key immune terms were identified for the calculation of the IRS. We further explored the clinical and genetic differences between the two IRS‐defined patient groups. Additionally, the IRS demonstrated its ability to predict the immunotherapy response in ccRCC patients.

**FIGURE 2:**
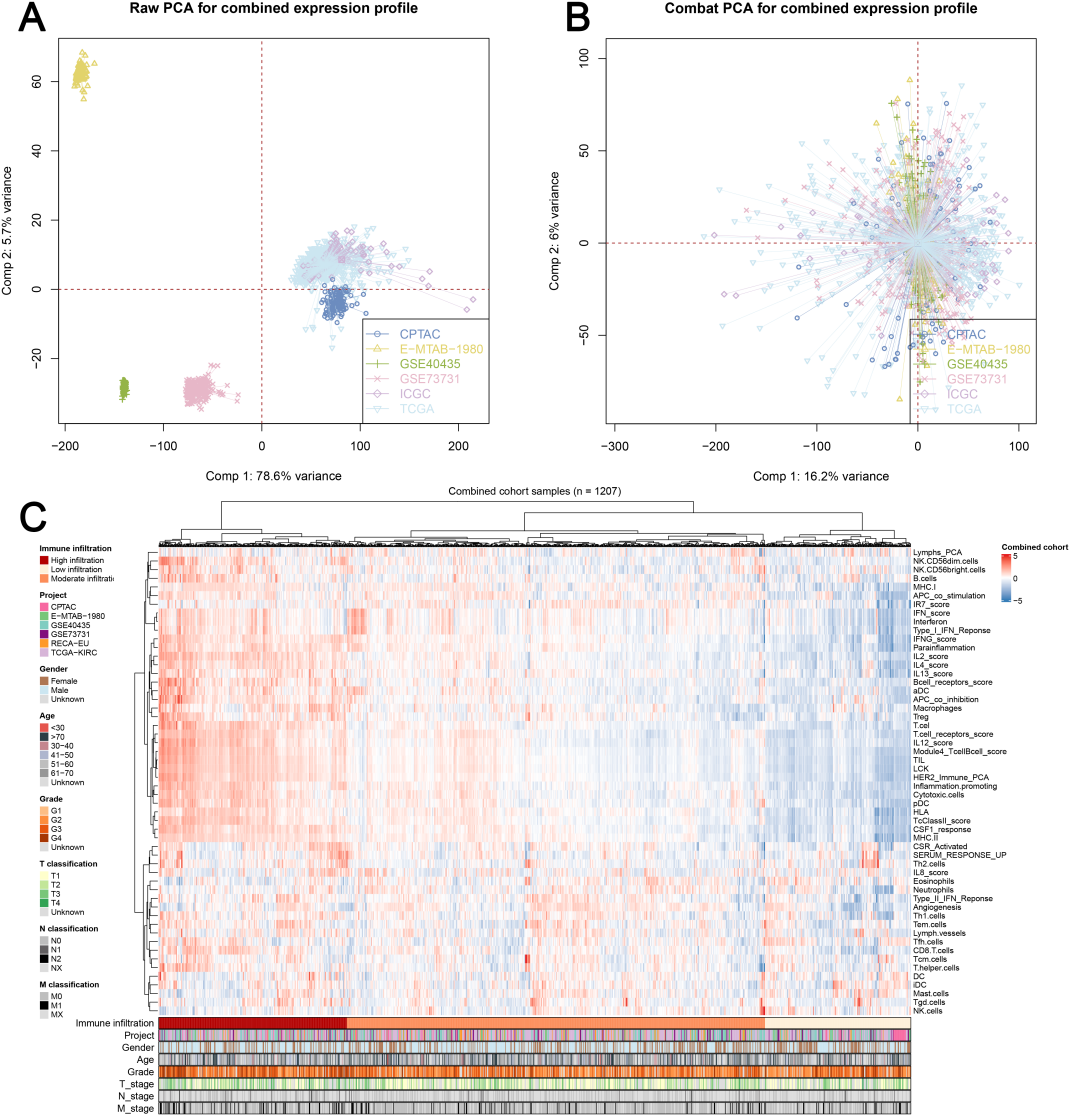
Combination of ccRCC cohorts and quantification of immune terms. (A) The six ccRCC cohorts selected for analysis exhibited significant differences; (B) batch difference of combination cohort was greatly reduced using R sva package; (C) each patient's expression matrix was evaluated for 53 immunological terms utilising R ssGSEA package. The heatmap displayed these immunological terms and the accompanying clinical characteristics of patients.

The study design's flowchart is depicted in Figure 1. After a comprehensive retrieval of multiple publicly available databases, six patient cohorts meeting our criteria, including TCGA, ICGC, E‐MTAB‐1980, CPTAC, GSE73731 and GSE40435, were selected for subsequent analysis. Batch effects were significant between the six cohorts (Figure 2A; Comp 1: 78.6% variance, Comp 2: 5.7% variance). Therefore, the ComBat function of the sva package was utilised to eliminate the possibility of batch effects (Figure 2B; Comp 1: 16.2% variance, Comp 2: 2.6% variance). Meanwhile, based on gene expression profiles, the ssGSEA package was used to identify the relative abundance of 53 immunological keywords (Figure 2C; 1207 samples).

### 3.2 Development of Immune Terms‐Based Prognostic Signature

**FIGURE 3:**
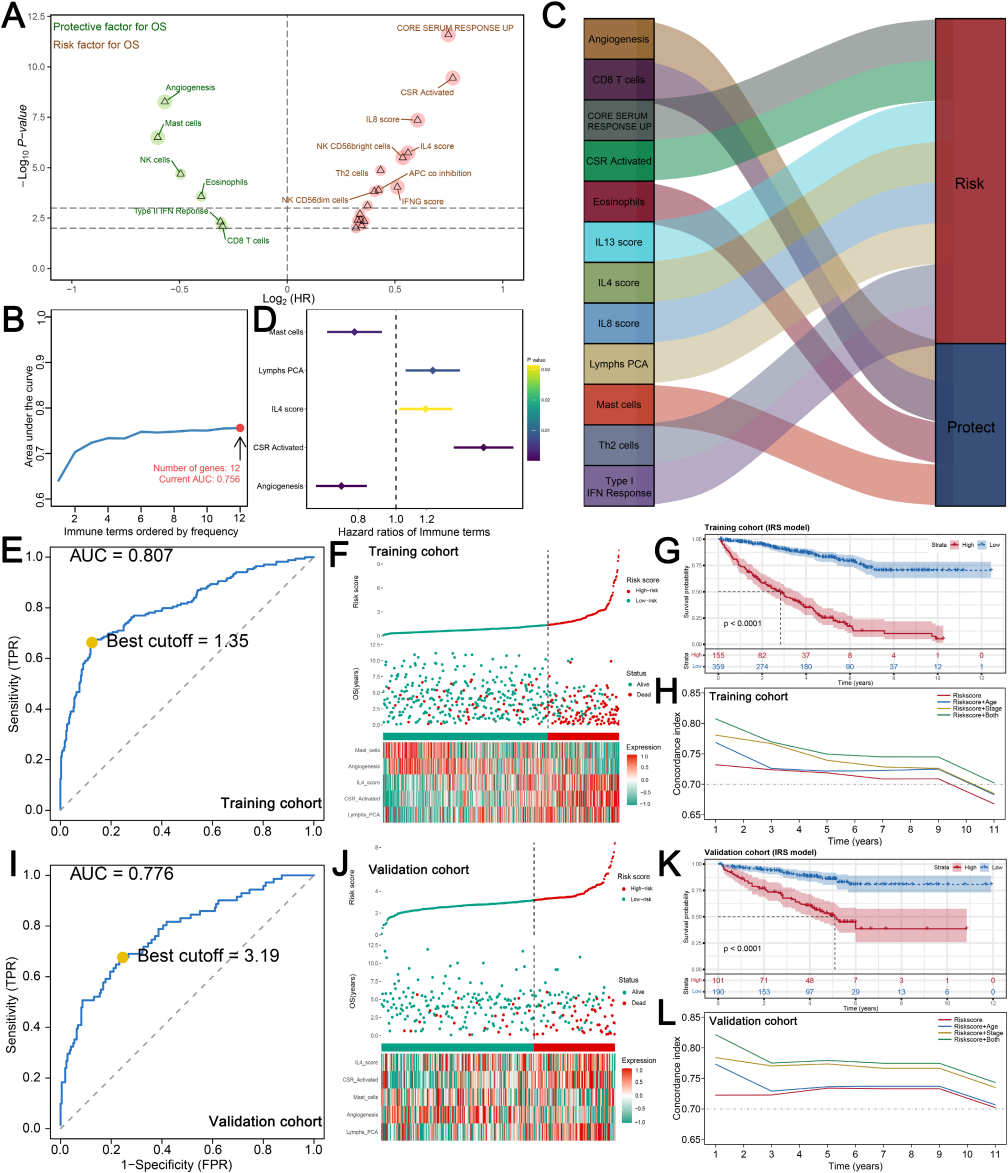
Identification of prognosis‐related immune terms and IRS calculation. (A) Six protective and sixteen risk immune terms related to prognosis were identified through univariate Cox analysis; (B) twelve immune terms with frequency > 50 were screened through L1‐penalised (lasso) estimation across 1000 iterations; (C) the integrated Sankey depicted the prognostic influence of these twelve immunological terms; (D) five were discovered for IRS calculation using multivariate Cox regression analysis; (E, I) the optimal IRS cutoff value was determined using the R survival package for both the training and validation cohorts; (F, J) patients with a high IRS had a greater mortality rate, as indicated by the risk plot (training cohort and validation cohort). (G, K) Kaplan–Meier curves showed that patients with a high IRS tended to have poorer prognoses in both cohorts; (H, L) the concordance index demonstrated that the combination of IRS, age and stage improved survival prediction accuracy in both the training and validation cohorts.

We selected patients with complete follow‐up information and a survival time of at least 30 days for further analysis. In the validation cohort, 53 immunological variables were analysed using univariate Cox regression; among these, 6 protective factors and 16 risk factors associated with prognosis were identified with *p*‐value < 0.01 (Figure 3A). For dimension reduction, a variable selection approach based on L1‐penalised (lasso) estimation was utilised, and 12 immunological terms with a frequency greater than 50 times were selected for multivariate Cox regression analysis (Figure 3B,C). Finally, a prognostic prediction signature was constructed using the formula: IRS = Angiogenesis* −0.322754412+CSR_Activated* 0.51830447+IL4_score* 0.175083671+Lymphs_PCA* 0.217838267+Mast_cells* −0.245207667 (Figure 3D). The optimal cutoff values for categorising patients as high or low IRS were determined using the SimDesign and tdROC packages, identifying 1.35 and 3.19 as the values with the highest AUCs of 0.807 and 0.776 in the training and validation cohorts, respectively (Figure 3E,I). The mortality rate of patients increased significantly with the increase in IRS in both the training and validation cohorts (Figure 3F,J), and KM survival analysis revealed that the high IRS group had a significantly shorter overall survival time compared to the low IRS group (Figure 3G,K). Furthermore, our analysis indicated that combining IRS with patient clinical information (age and stage) can improve the predictive performance of the model (Figure 3H,L).

### 3.3 Significant Discrepancy of Clinical Correlation and Biological Function

**FIGURE 4:**
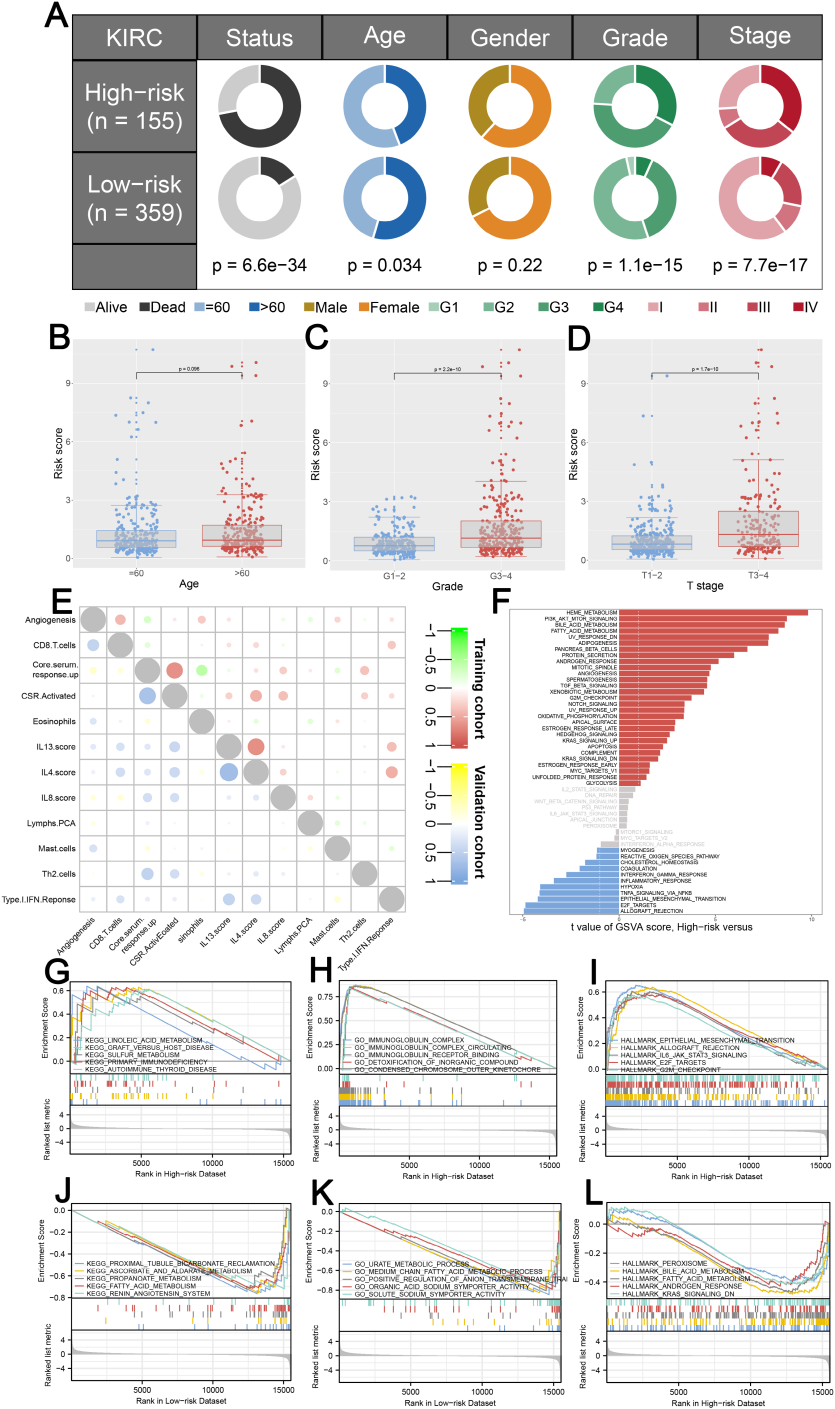
Correlation between IRS and clinical features of ccRCC patients. (A) Pie charts illustrating the Chi‐squared test for clinicopathologic characteristics comparing two IRS groups in KIRC; (B–D) correlation analysis between IRS and clinical factors, including age, grade and T stage; (E) Co‐expression relationship between five signature immunological terms; (F) GSVA analysis comparing the two IRS groups, with upregulated pathways in high IRS patients shown in red and those in low IRS patients shown in blue; (G‐L) GSEA analysis of the signature depending on annotated gene set files.

Patients with high IRS may experience adverse events and have worse baseline characteristics (Figure 4A). We discovered no association between IRS and age, but a significant correlation with grading and T stage, indicating that patients with higher grades or more advanced pathological stages tend to have higher IRS (Figure 4B–D). A correlation coefficient heatmap was used to depict the co‐expression relationships among distinct immunological terms (Figure 4E). Moreover, the most significantly upregulated pathways in the GSVA among high IRS patients included heme metabolism, PI3K/AKT/mTOR signalling, bile acid metabolism, fatty acid metabolism and UV response. In contrast, allograft rejection, E2F targets, epithelial‐mesenchymal transition (EMT), TNFa signalling via NFkB and hypoxia were aberrantly activated in low IRS patients (Figure 4F). Further biological processes and pathways enrichment analysis between the two IRS groups revealed significant discrepancies in the GO, KEGG and Hallmark pathways (Figure 4G–L).

### 3.4 CPA4 Promotes ccRCC Cell Proliferation, Migration and Invasion

**FIGURE 5:**
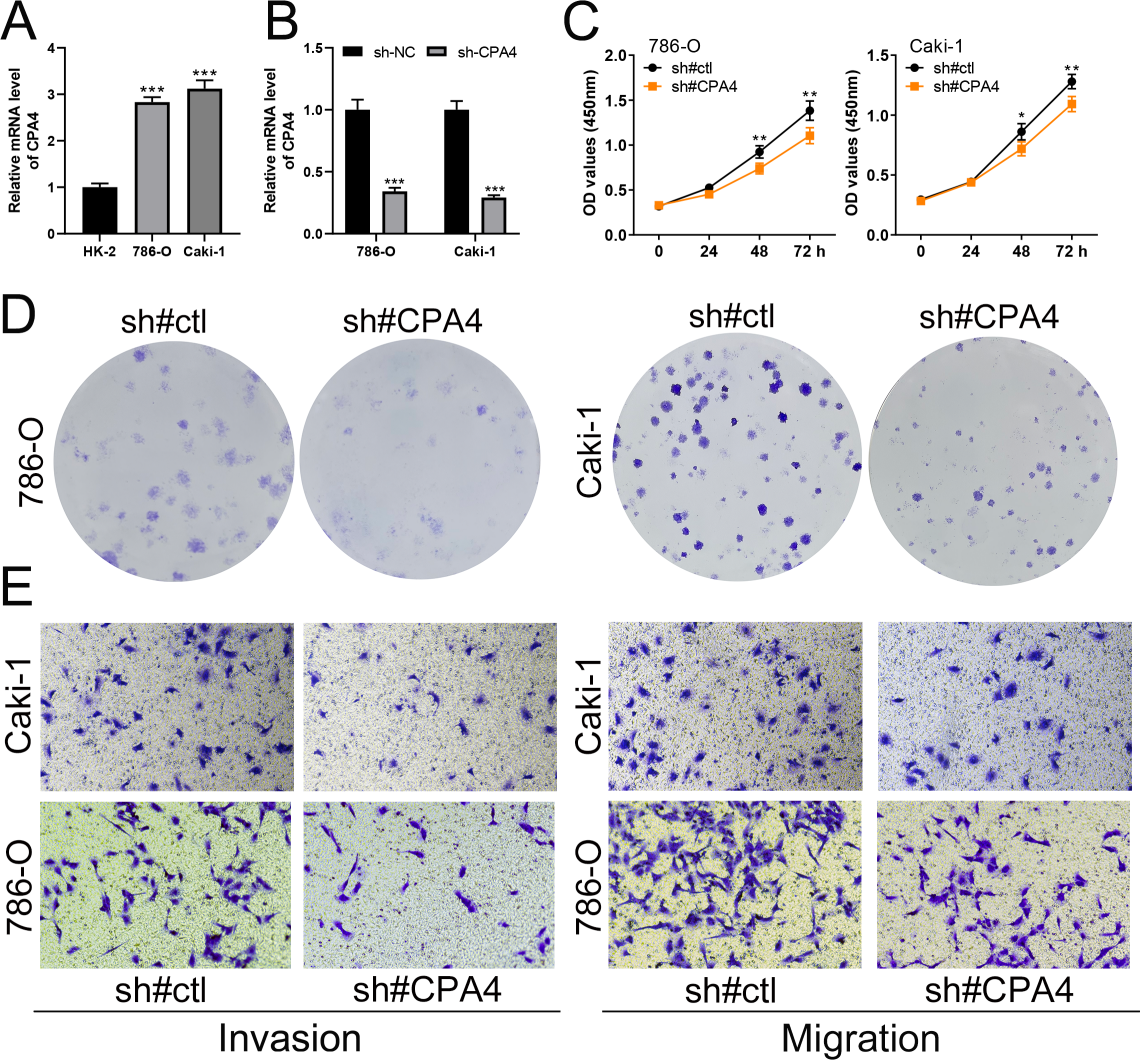
CPA4 promotes ccRCC cell proliferation, migration and invasion. (A) CPA4 expression in HK2, 786‐O and Caki‐1 cell lines as measured by qPCR; (B) CPA4 knockdown efficiency in 786‐O and Caki‐1 cells, showing significant reduction of CPA4 expression; (C) CCK‐8 assays showing reduced cell proliferation following CPA4 knockdown in 786‐O and Caki‐1 cells; (D) colony formation assays demonstrating decreased colony formation ability in CPA4‐knockdown cells; (E) transwell migration and invasion assays showing reduced migratory and invasive capabilities in CPA4‐knockdown cells. (* = *p* < 0.05, ** = *p* < 0.01, *** = *p* < 0.001).

Based on the pathway enrichment analysis, we selected CPA4 for further investigation due to its highest logFC among differentially expressed genes, suggesting a significant role in ccRCC progression. We first assessed CPA4 expression levels in human kidney epithelial cell line HK2 and ccRCC cell lines 786‐O and Caki‐1. CPA4 was markedly upregulated in both 786‐O and Caki‐1 cells compared to HK2 (Figure 5A). To explore its functional role, we established stable CPA4 knockdown cell lines in 786‐O and Caki‐1, confirming efficient CPA4 suppression by qPCR (Figure 5B). Knockdown of CPA4 led to a significant reduction in cell proliferation, as demonstrated by lower OD values in CCK‐8 assays (Figure 5C). Similarly, colony formation assays revealed a marked decrease in clonogenic potential in CPA4‐knockdown cells, suggesting impaired growth capacity (Figure 5D). Furthermore, Transwell assays indicated that CPA4 knockdown reduced the migration and invasion abilities of 786‐O and Caki‐1 cells (Figure 5E). These findings strongly support the oncogenic role of CPA4 in ccRCC by promoting cell growth, migration and invasion.

### 3.5 Comparison of Genomic Characteristics Between Two IRS Groups

**FIGURE 6:**
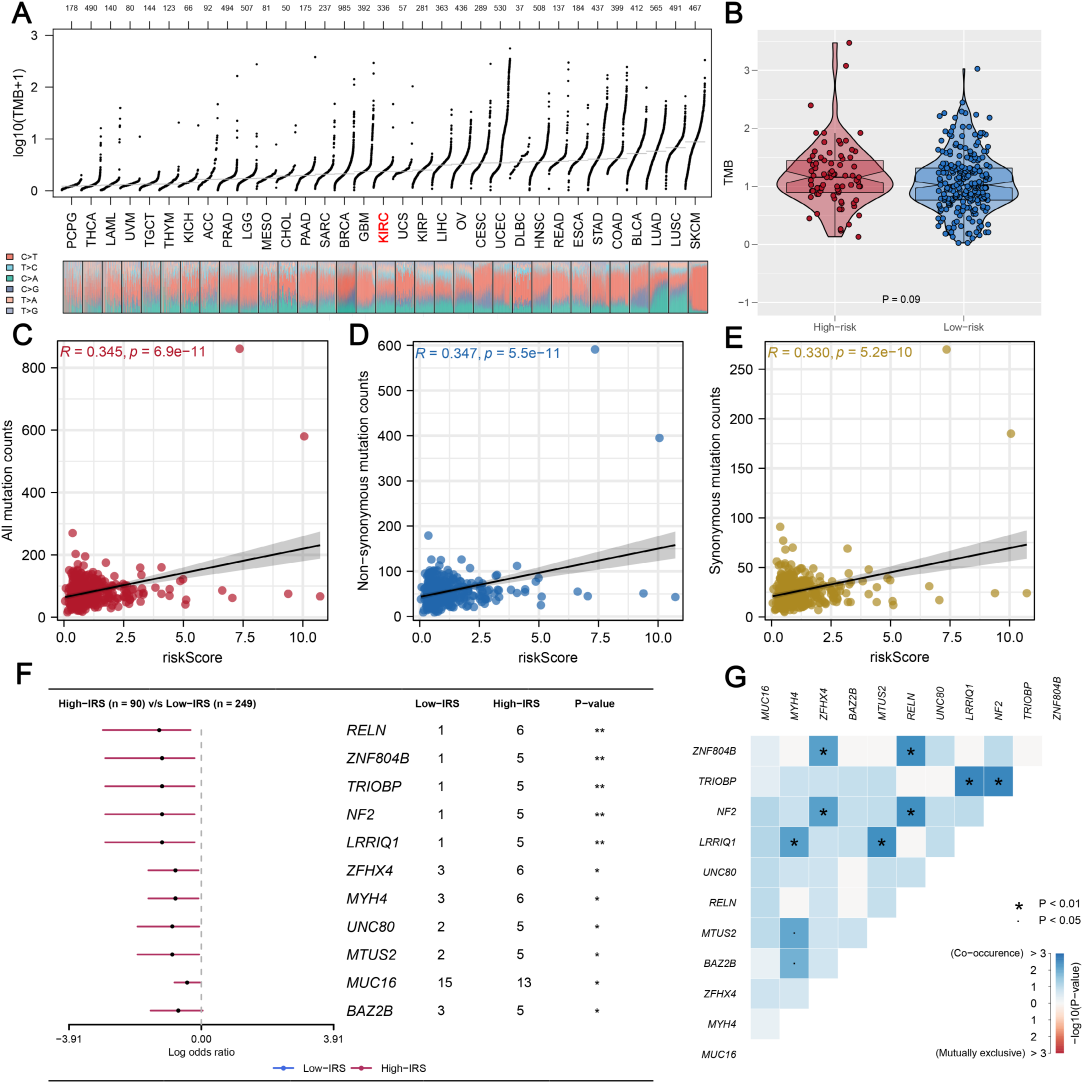
Correlation of IRS with tumour mutational burden (TMB). (A) Overview of TMB distribution in TCGA pan‐cancer; (B) patients with high IRS had a higher TMB concentration; (C–E) the correlation between IRS and genome mutation counts including all mutation counts, non‐synonymous mutation counts and synonymous mutation counts; (F) forest plot of genes mutating differentially in patients of both two groups. (G) Co‐mutation relationship of mutation genes. (* = *p* < 0.05, ** = *p* < 0.01, *** = *p* < 0.001).

**FIGURE 7:**
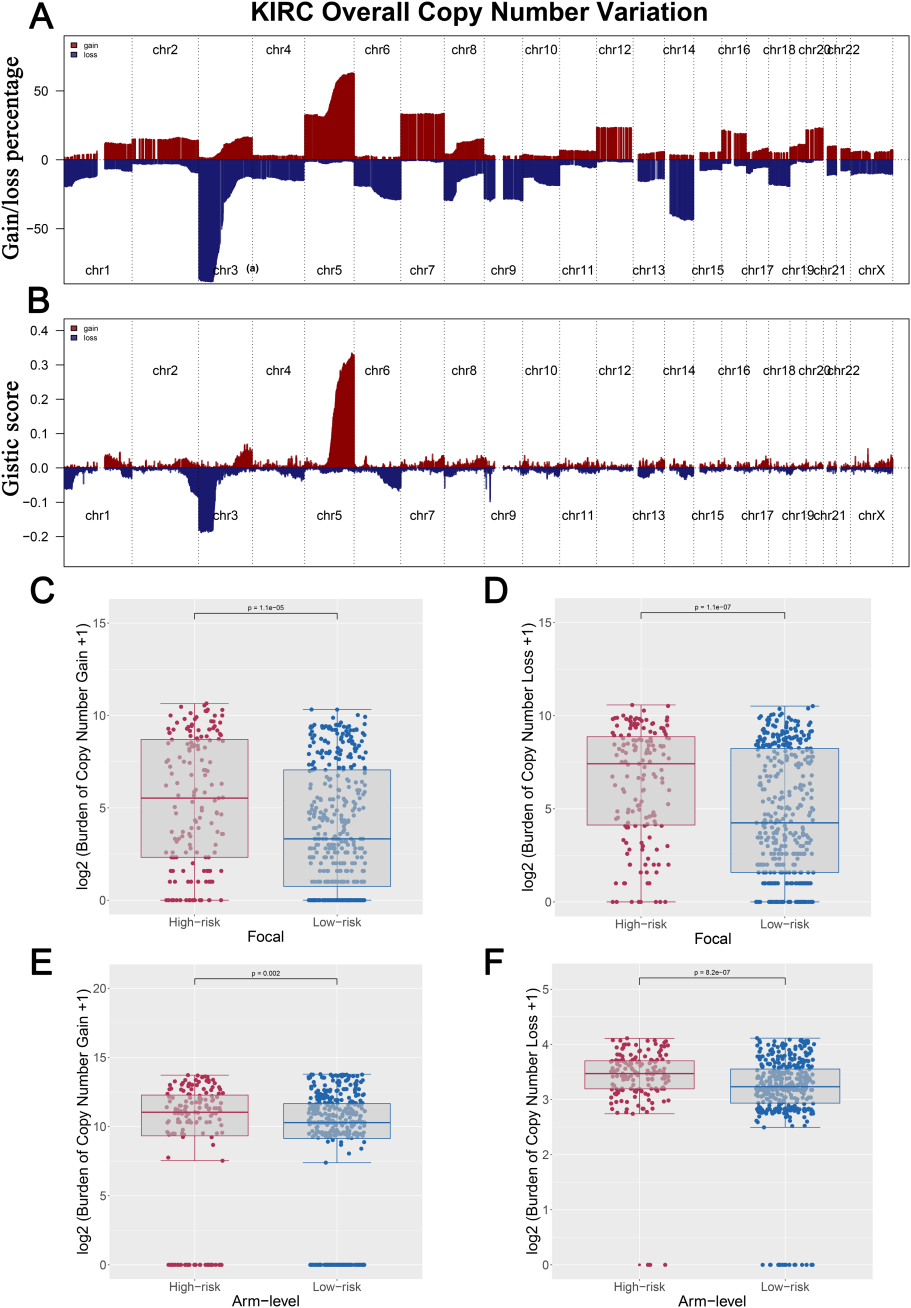
Copy number variation (CNV) partition across two IRS groups. (A) The percentage of CNV gains and losses in TCGA‐KIRC; (B) the gistic score of CNV of TCGA‐KIRC; (C–D) patients with a higher IRS exhibited a higher focal CNV burden; (E–F) Patients with high IRS had a higher arm level of CNV burden.

**FIGURE 8:**
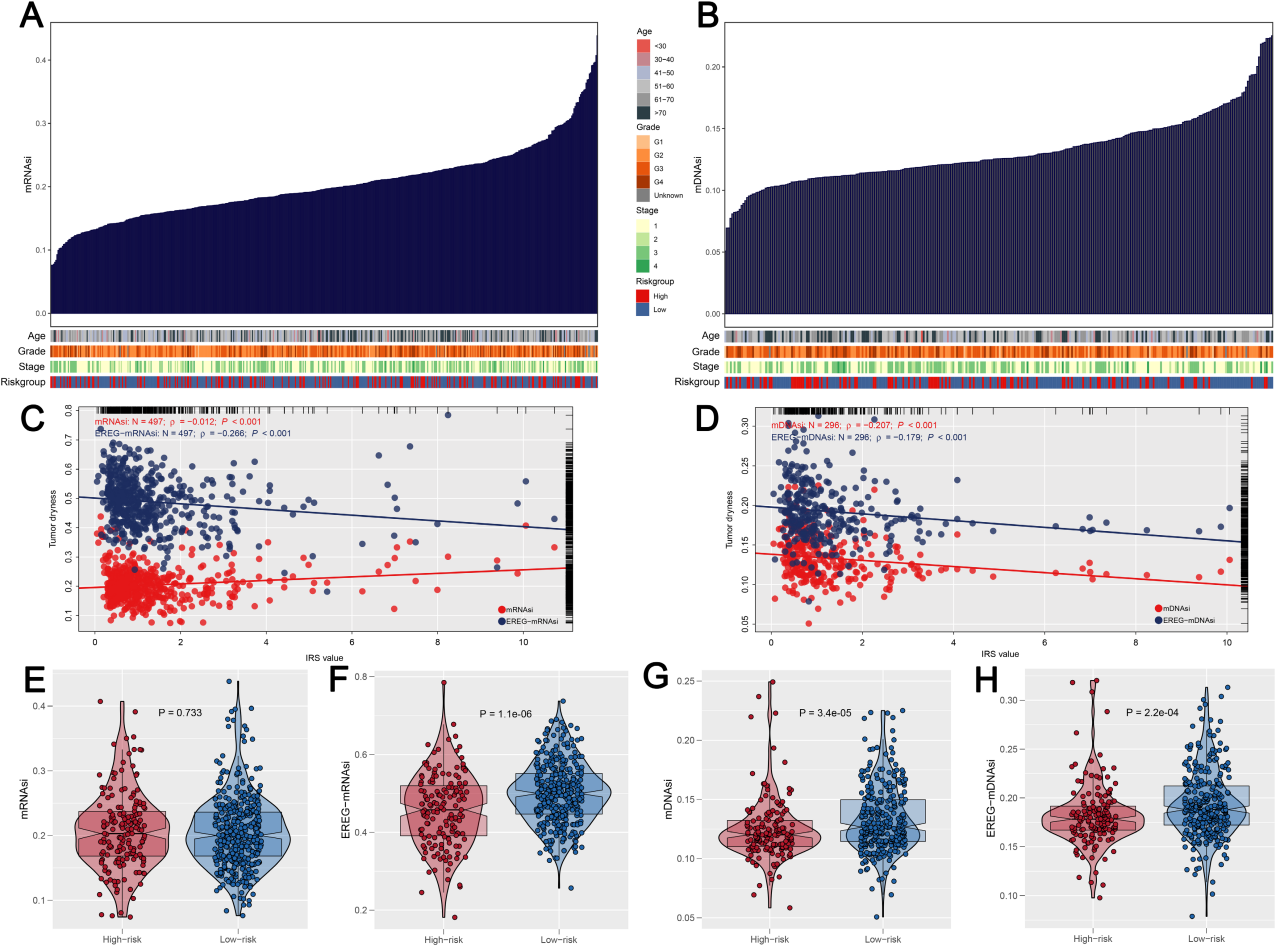
Correlation of IRS with tumour stemness indices. (A, B) Overview of the relationship between known clinical and molecular features (age, grade, stage and IRS) and stemness indices (mRNAsi and mDNAsi); (C, D) IRS was negatively related to tumour stemness indices (mRNAsi, EREG‐mRNAsi, mDNAsi and EREG‐mDNAsi); (E–H) patients with low IRS exhibited higher levels of tumour stemness indices (mRNAsi, EREG‐mRNAsi, mDNAsi and EREG‐mDNAsi).

**FIGURE 9:**
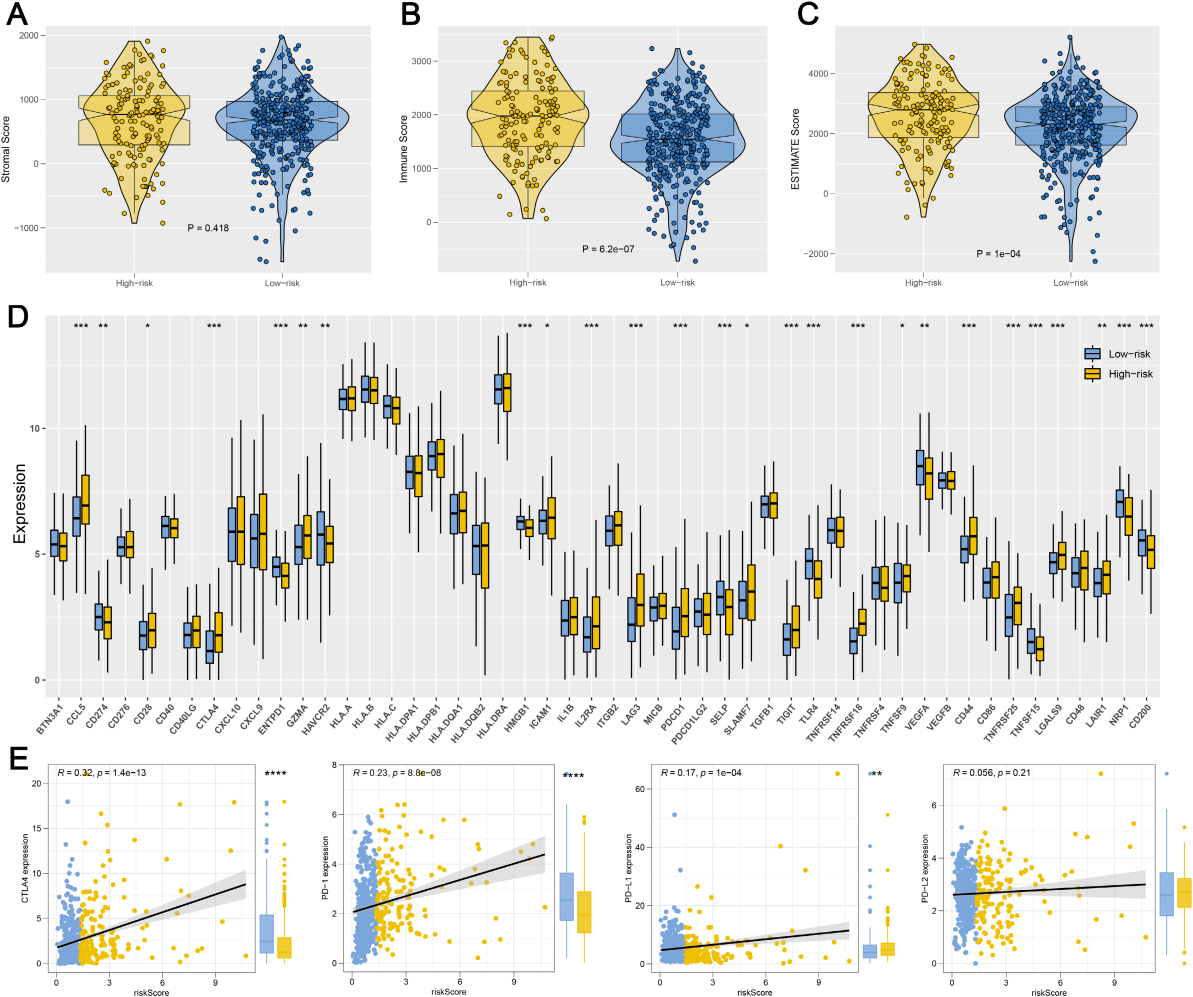
Exploration of the difference of immune features and immune checkpoint genes between two IRS groups. (A–C) Patients with high IRS showed a higher level of immune score, stromal score and estimate score. (D) Comparison of expression of multiple immune checkpoint genes between two IRS groups; (E) patients with high IRS exposed increased levels of CTLA4 and PD‐1. (* = *p* < 0.05, ** = *p* < 0.01, *** = *p* < 0.001).

First, using data from the TCGA database, we evaluated tumour mutational burden (TMB) across various cancer types and observed that ccRCC patients generally exhibited lower TMB levels compared to other cancers (Figure 6A). When comparing TMB between the two IRS groups within the ccRCC cohort, no significant differences were identified (Figure 6B). However, further analysis revealed that IRS was positively correlated with the overall number of genomic mutations, including both total mutation counts and specific mutation types such as synonymous and non‐synonymous mutations (Figure 6C–E). This suggests that while TMB itself may not differentiate between IRS groups, the broader spectrum of mutations is more prevalent in patients with higher IRS scores. Additionally, we identified a set of genes—RELN, ZNF804B, TRIOBP, NF2, LRRIQ1, ZFHX4, MYH4, UNC80, MTUS2, MUC16 and BAZ2B—that were more frequently mutated in the high IRS group (Figure 6F). These mutations appeared to co‐occur, indicating potential mutational synergies or shared pathways contributing to the aggressive nature of tumours in patients with elevated IRS (Figure 6G). Secondly, CNV in ccRCC patients, including the percentage of gain/loss CNV and GISTIC score, were analysed (Figure 7A,B). The CNV analysis showed that patients with a high IRS exhibited a greater incidence of focal and extensive CNV than those with a low IRS (Figure 7C–F). Thirdly, since cancer stem cells (CSCs) are suspected to be the source of tumour survival and chemotherapy resistance, we ranked ccRCC patients based on their mRNAsi or mDNAsi levels (from low to high stemness index) to understand the differences in the tumour microenvironment (Figure 8A,B). We found that the stemness index had a negative association with IRS (Figure 8C,D), and patients in the high IRS group were found to have a lower stemness index than those in the low IRS group (Figure 8E–H). Finally, we also found that patients in the high IRS group exhibited greater immunological scores, estimation scores and stromal scores than those in the low IRS group (Figure 9A–C). Furthermore, given the importance of immunological checkpoints in tumour development, we studied the relationship between IRS and various checkpoints (Figure 9D), and the results showed that patients with a low IRS had lower levels of CTLA4 and PD‐1 (Figure 9E), implying that the immunotherapy response may differ between the two IRS patient groups.

### 3.6 Immunotherapeutic and Targeted Therapy Response Prediction Based on IRS

**FIGURE 10:**
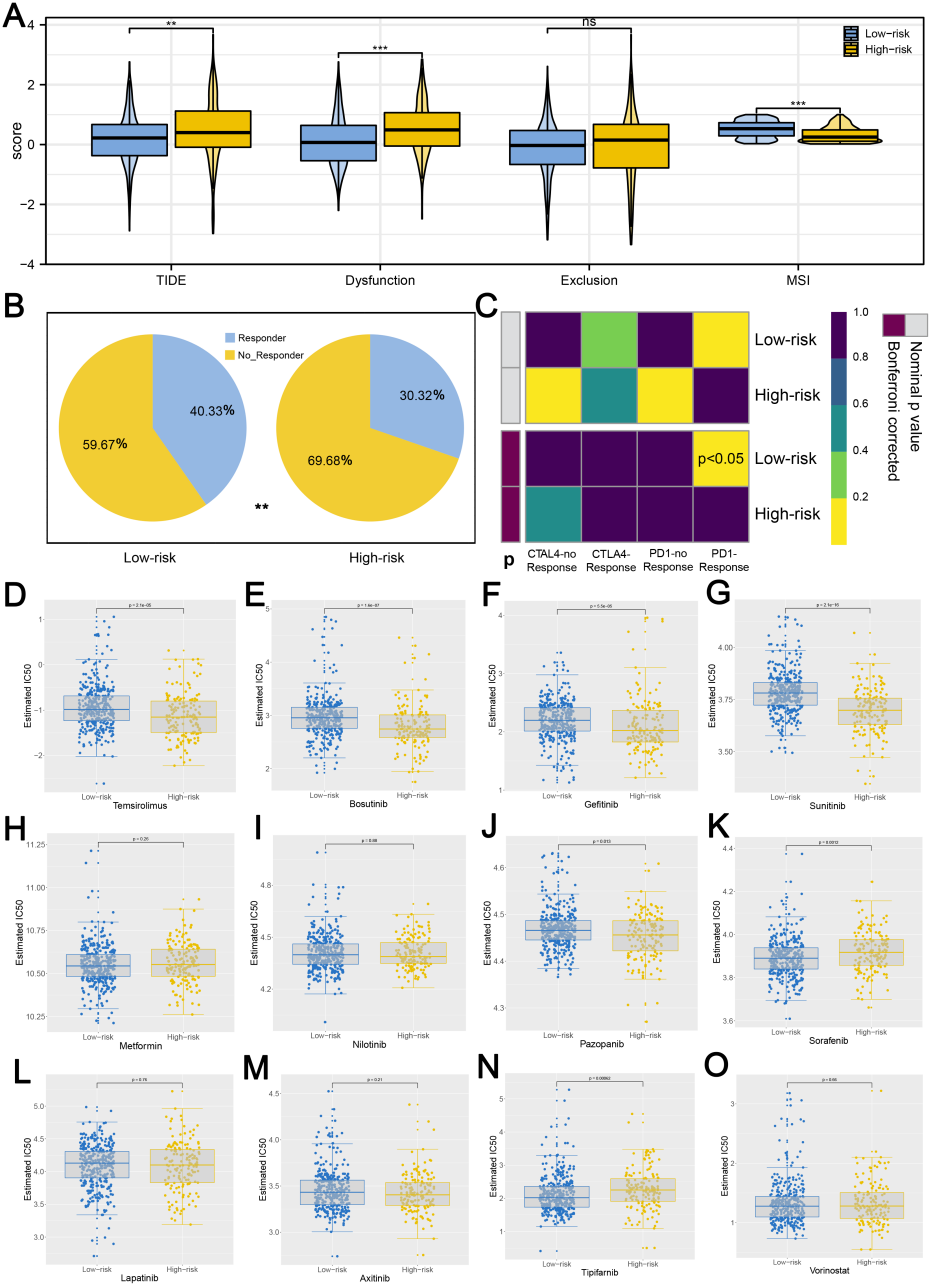
Immunotherapeutic and chemotherapeutic response prediction between two IRS groups. (A) Comparisons of TIDE score for chemotherapeutics and targeted therapy of the signature. (B) Patients with low IRS have a greater percentage of responders (TIDE score < −0.2 were considered as responders and TIDE score > 0.2 were considered as non‐responders). (C) Subclass mapping analysis manifested patients with low IRS might be more sensitive to the PD‐1 inhibitor (Bonferroni corrected *p*‐value = 0.043). (D–O) The box plots of the estimated IC_50_ for 12 commonly used chemotherapy. (* = *p* < 0.05, ** = *p* < 0.01, *** = *p* < 0.001).

TIDE analysis, considered a more accurate biomarker for determining the probability of immunotherapeutic response, was conducted to investigate the link between immunotherapeutic response and IRS. The results revealed that IRS‐low patients had a lower TIDE score (Figure 10A). A TIDE score > 0.2 was classified as non‐responders, whereas a TIDE score < −0.2 was identified as responders. More responders were found in the low IRS group than in the high IRS group, suggesting that patients in the low IRS group are more likely to respond to immunotherapy (Figure 10B). In addition to TIDE predictions, we performed subclass mapping analysis to investigate the expression similarity between the two IRS groups and patients who responded positively to immunotherapies. Correspondingly, we were pleased to discover that low IRS patients were more likely to be PD‐1 immunotherapy responders (Figure 10C). Meanwhile, we used the Genomics of Drug Sensitivity in Cancer (GDSC) to estimate the IC50 of 12 targeted therapy drugs for each patient between the two IRS groups (Figure 10D–O).

## 4 Discussion

ccRCC, well‐known for its high metastasis and relapse risk, is responsible for the majority of RCC‐related mortality [17]. As one of the tumours long known as ‘immunotherapy‐responsive’, ccRCC stands out in pan‐cancer comparisons for having one of the highest immune infiltration rates [18]. In the early stages, the only curative therapy for patients remains conventional radical nephrectomy or partial nephrectomy. However, most patients are diagnosed at advanced stages, and the recurrence or metastasis rate for those undergoing radical nephrectomy is still higher than 20% [19]. Currently, the evolution of targeted therapy and immunotherapy has filled the gap in the treatment of ccRCC, with PD‐1 inhibition‐based therapy being a breakthrough for advanced malignancies, especially ccRCC [20, 21]. In this investigation, a predictive signature based on immunological parameters was established that can effectively predict the prognosis of ccRCC patients, as well as reliably predict responses to targeted therapy and immunotherapy.

Here, we first combined six different ccRCC cohorts into a large population cohort and categorised them into 53 immunological terms. We then identified 6 protective immune terms and 16 risk immune terms related to prognosis using univariate Cox regression analysis. Five immune terms, including mast cells, lymphs PCA, IL4 score, CSR activated and angiogenesis, were finally selected for IRS calculation based on 1000 iterations of L1‐penalised estimation and multivariate Cox regression. Almost all tumour types have an elevated density of mast cells, which are primary components of the tumour immunological environment [22]. Additionally, mast cells have been shown to be therapeutic targets for solid malignancies such as ccRCC [7]. Meanwhile, a systematic review indicated that lymph angiogenesis and lymph node invasion play significant roles in tumour metastasis, with peritumoral lymphatics (PTLs) being more important for urothelial carcinoma metastatic cells than intratumoral lymphatics (ITLs) [23]. IL4, known to be capable of activating tumours, has also been shown to be associated with an increased risk of developing chronic renal disease [24, 25]. Furthermore, angiogenesis is a crucial aspect of tumour growth and metastasis, interacting not only with tumour cells but also with immunological cells, fibroblasts, pericytes and the extracellular matrix (ECM) [26]. Advances in tumour immunotherapy may make it possible to examine the immune environment of ccRCC in more detail now that the crucial immune terms in our study have been identified.

Furthermore, we performed clinical correlation analysis, and the data revealed that higher IRS is related to worse clinical outcomes. GSEA also showed significant biological differences between the two IRS groups: EMT, JAK/STAT pathway and G2/M checkpoint were upregulated among higher IRS patients. It is widely believed that EMT plays a role in tumorigenesis and tumour progression. Xia et al. [27] reported that the progression of RCC was inhibited by suppressing the EMT process. One of the most commonly deregulated pathways in cancer is JAK/STAT signalling, and studies have revealed that ccRCC is primarily caused by the abnormal activation of the JAK/STAT pathway [28]. The G2/M checkpoint, known as a DNA‐damage checkpoint in cell cycle regulation, can suppress the proliferation of cells. In the study on colon cancer cells, Roy et al. [29] showed that apoptotic cell death was linked to G2/M phase arrest. Conversely, KRAS signalling was downregulated in low IRS patients. Our findings suggest that abnormal activation of the aforementioned pathways may lead to poorer clinical and genomic outcomes among high IRS patients.

High genomic instability contributes to tumour cell killing through the immune system, which can impact immunotherapy response. Liu et al. [30] revealed that the combination of TMB and CNV could accurately predict prognosis and the efficacy of ICI therapy. Patients with a high level of TMB and a low level of CNV showed favourable responses to ICI therapy, which is consistent with our results. Meanwhile, we found that RELN, ZNF804B, TRIOBP, NF2, LRRIQ1, ZFHX4, MYH4, UNC80, MTUS2, MUC16 and BAZ2B were the most frequently mutated genes in the high IRS group. Quantification of tumour stemness reported that low IRS patients have a higher stemness index compared with patients in the high IRS group. Moreover, the expression of many widely used immunological checkpoints, such as PD‐1 and CTLA4, was significantly linked to elevated IRS, which may relate to the response rate of immunotherapy. All findings demonstrated that IRS is a reliable indicator of therapeutic response.

TIDE analysis was applied to validate our findings, and the results showed that patients with a low IRS had a lower TIDE score and a higher MSI score, implying that they have a greater immunotherapy response rate. Furthermore, we also used the subclass mapping algorithm to evaluate similarities between patients who responded to immunotherapies and patients in the two IRS groups. Consistent with the TIDE analysis, patients with a low IRS had a greater likelihood of responding to immunotherapy. Moreover, since targeted therapy is a common approach for treating ccRCC, we performed an analysis using the GDSC dataset, and the results showed significant differences in the estimated IC50 of multiple commonly used targeted therapy agents. Notably, patients with a high IRS had a lower IC50 for sunitinib, a first‐line targeted therapy drug for ccRCC patients, indicating that high IRS patients may respond better to certain targeted therapies. While the IRS demonstrated strong potential for predicting prognosis and guiding treatment responses in ccRCC, its practical application in clinical settings requires further validation. For example, the IRS could be employed to stratify patients into different treatment categories, helping clinicians decide who may benefit more from ICIs or targeted therapies like sunitinib.

There are inevitably some limitations that need to be noted and should be addressed in future studies. Firstly, we mainly used samples from Western populations, which might introduce bias in population and genomic analysis. Secondly, although most patients in our study had complete prognosis information, we lacked detailed clinical features such as lifestyle. Additionally, further studies are needed to evaluate how the IRS applies across different stages of ccRCC and interacts with other clinical factors. Future research should also explore the mechanistic roles of individual immune terms to better understand their contributions to ccRCC progression and treatment. Despite these limitations, we effectively developed and validated a strong predictive signature linked with immunotherapy response in both training and validation cohorts with large sample sizes.

## 5 Conclusion

In conclusion, this study established a prognostic signature based on immune terms in a large ccRCC cohort and investigated the clinical relevance and biological functions of patients between two IRS groups. Further analysis revealed that patients with high IRS had worse clinical characteristics and prognoses. TMB, CNV, tumour stemness index and TIDE analysis showed significant discrepancies in tumour genomic features between the two IRS groups, indicating that immunotherapy response was more frequent in patients with a low IRS.

## Author Contributions

M.P. supervised the project design. X.X. handled data collection. D.Z. performed data analysis and wrote the initial manuscript. W.C. reviewed and edited the manuscript.

## Ethics Statement

The authors have nothing to report.

## Consent

All authors have reviewed and approved the final version of the manuscript and consent to its publication.

## Conflicts of Interest

The authors declare no conflicts of interest.

## Data Availability

All the data used in our study are included in the article, and further inquiries can be directed to the corresponding author.
